# Mitral Valve Surgery in Patients with Systemic Lupus Erythematosus

**DOI:** 10.1155/2014/216291

**Published:** 2014-10-14

**Authors:** Mahnoosh Foroughi, Manouchehr Hekmat, Mohsen Ghorbani, Hamid Ghaderi, Masoud Majidi, Mahmood Beheshti

**Affiliations:** Cardiovascular Research Center, Shahid Beheshti University of Medical Sciences, Modarres Hospital, Saadat Abad, Tehran 19987 34383, Iran

## Abstract

Valvular heart disease is the common cardiac manifestation of systemic lupus erythematosus (SLE) with a tendency for mitral valve regurgitation. In this study we report a case of mitral valve replacement for mitral stenosis caused by Libman-Sacks endocarditis in the setting of SLE. In addition, we provide a systematic review of the literature on mitral valve surgery in the presence of Libman-Sacks endocarditis because its challenge on surgical options continues. Surgical decision depends on structural involvement of mitral valve and presence of active lupus nephritis and antiphospholipid antibody syndrome. Review of the literature has also shown that outcome is good in most SLE patients who have undergone valvular surgery, but association of antiphospholipid antibody syndrome with SLE has negative impact on the outcome.

## 1. Introduction

Most parts of the heart may be affected in the course of systemic lupus erythematosus (SLE): coronary arteries, valves, conduction system, pericardium, myocardium, and endocardium, with a variety of clinical manifestations, and it is the main cause of morbidity and mortality in these patients [1–3]. Valvular involvement is the frequent cardiac manifestation in SLE. It has been shown that the prevalence of valvular disease (diagnosed by echocardiography) in SLE can be more than 50% [4–6].

Libman-Sacks (LS) endocarditis was first described in 1924 by Libman and Sacks, as the cardiac manifestation in SLE and antiphospholipid antibody syndrome (APS) [1, 5, 7]. It is noninfectious verrucous vegetative lesions that may clinically mimic infective endocarditis. Involvement of all four valves could be seen in SLE patients. It has a preference on the left side of the heart, mainly on the mitral valve, followed by the aortic valve, but it also can be seen on the papillary muscle and chordae tendinae, left atrial wall, left ventricular septum, and aortic wall. Mitral regurgitation and aortic insufficiency are the prominent functional abnormality, although stenotic aortic and mitral valve have been rarely reported. In right-sided valves, thickening and vegetation are more seen in tricuspid valve than pulmonary and there is a low prevalence of pulmonary hypertension in SLE patients [8, 9]. The valve involvement stimulates a broad spectrum of presentations. It is typically mild and asymptomatic but can lead to fulminant presentation such as superimposed bacterial endocarditis, thromboembolic events, and congestive heart failure [1, 2, 5, 7]. Echocardiography remains the best imaging modality for early and accurate diagnosis and helps to avoid misdiagnosis (papillary fibroelastoma and infective endocarditis). The definitive diagnosis can be achieved by histopathologic examination of the valve [1–5]. It consists of fibrin deposits, fibroblastic organization, neovascularisation, immune complexes, and infiltration of mononuclear cells [1, 10]. In the healed form, there is a fibrous plaque with focal calcification but marked scarring and valve deformity is seen in the presence of extensive involvement in the other extreme of the disease, which may require surgical treatment (repair/replacement). It was suggested that the presence of LS endocarditis has correlation to longer duration of disease, higher disease activity, and increased risk of thrombotic events [1, 2, 7, 11]. However, heart failure (due to mitral regurgitation) as the first manifestation of the disease was reported too [12].

Data regarding the outcome of mitral valve surgery in patients with SLE are limited and generally restricted to case reports or small series. In this study we report a case of mitral valve replacement for severe stenosis caused by LS endocarditis. In addition, we provide a systematic review of the literature for surgical consideration and highlight some important concerns that need to be addressed.

## 2. Case Presentation

A 17-year-old girl presented at our institution with longstanding SLE. The first presentation was macroscopic hematuria when she was 2 years old. The definitive diagnosis was made by renal biopsy 9 years ago and she has been treated since then. The patient was admitted with progressive exertionaldyspnea and unexplained tachycardia of 2-month duration. Ten months prior to this episode she had developed central facial paresis and pancytopenia. She had no history of rheumatic fever. Echocardiography revealed severe mitral stenosis with thickened mitral valve leaflets, small vegetations on the posterior mitral valve leaflet, and chordae, with no pleural and pericardial effusion.

Her medications were CellCept (mycophenolatemofetil, 500 mg/12 h) and oral steroid (prednisolone, 7.5 mg/daily). Admission laboratory tests revealed normal serum chemistry.

The surgery was performed using standard cardiopulmonary bypass with cardioplegic arrest and intermittent cold bloody cardioplegia. Heparin was administered at initial dose of 300 IU/kg and additional dose was repeated to maintain activated clotting time >450 seconds. There were no dense pericardial and pleural adhesions. Both the anterior and posterior mitral leaflets were thickened, fibrotic with diffuse focal vegetations, but the annulus was intact and normal. Vegetations extended to the subvalvular apparatus. The bands of tissue involved most of the chordae of the posterior mitral leaflet. Therefore, mitral valve repair was not considered feasible and appropriate. The mitral valve was replaced with a 23 mm St. Jude aortic mechanical prosthesis in a reversed direction.

Pathological examination of the excised mitral valve leaflets showed fibrosis, neovascularization, vegetation with fibrin-platelet thrombi, and inflammatory cell infiltration which was consistent with LS endocarditis. The attached chordae tendineae were partially fused and myxomatous with focal fibrosis. The patient's recovery from surgery was uneventful and she was discharged on the seventh postoperative day.

## 3. Search Strategy

We reviewed the literature on mitral valve surgery in the SLE setting ± APS. Although valve thickening and vegetation (LS endocarditis) were described in SLE patients at first, it is the most common finding in APS patients too [1, 5, 27, 31]. Because of higher prevalence of valve involvement in APS secondary to SLE (more than 50%), this association is included in this search [1, 7, 17].

Medline and Google Scholar from 1970 through December 2013 used the PubMed interface: (title/abstract) systemic lupus erythematosus OR SLE OR Libman-Sacks endocarditis AND mitral valve surgery OR mitral valve replacement OR mitral valve involvement, without language restriction. We selected all relevant papers that related to mitral valve surgery (repair or replacement, due to regurgitation and/or stenosis, and ± other cardiac valve involvement) in the context of SLE and/or APS secondary to SLE. There were 19 case reports and 3 review articles identified in the world medical literature [5, 16, 17]. They were reviewed to suggest important applicable point for future use with surgically oriented option. Bouma et al. had selected only isolated mitral regurgitation (MR) caused by LS endocarditis in English journals in their systematic review [5]. We included all mitral valve pathology (regurgitation, stenosis, or both) that needed surgery in SLE patients ± APS, without language limitation. Gorki et al. reviewed mitral and aortic valve involvement in APS patients ± SLE [17]. Due to our focus on SLE disease, we selected SLE patients ± APS with mitral valve pathology. Hakim et al. appraised mitral surgery in SLE patients till 2001 [16].

There were 47 cases of pure MR and 17 cases of mitral stenosis (MS) reported in SLE patients with surgical treatment in the literature. Valve pathology was not defined in 15 patients. Tables 1 and 2 show that the surgical options for MS were less frequent in recent years. It is unknown whether advanced medical treatment and closer control would decrease grade of stenosis requiring surgery. Also five SLE patients that had both MS and MR were reported. This combination is more frequent in SLE with secondary APS.  Table 1 displays 18 cases of mitral repair; 4 of them needed reoperation during short follow-up. Mitral stenosis was the dominant pathology in these patients and valve calcification was the main cause of reoperation and valve replacement. None of them had APS association. It seems that the success rate of mitral valve repair in SEL patients is less than other etiologies and surgical repair for MS probably needs more reoperation than MR valves.

## 4. Discussion

According to the literature, the prevalence of cardiovascular involvement in patients with SLE has been estimated to be more than 50% [1, 4, 5, 16, 17]. It was shown that the left-sided heart valves are affected most commonly. The most frequently involved valve is the mitral valve followed by the aortic valve and regurgitation represents the predominant abnormality [1, 2, 4, 6, 9]. The mitral valve involvement in patients with SLE has been categorized as leaflet thickening, vegetations (LS endocarditis), regurgitation, and valve stenosis [1, 2, 5, 7–11, 16, 32]. Mitral stenosis is the least valvular manifestation (<5%) and is frequently associated with MR [4, 9].

It is thought that immunologic insults (infiltration of inflammatory cells and immune complex deposition) play principal role. There is the sequence of insult events from formation of fibrin-platelet thrombi, its organization, fibrosis, edema, diffuse thickening, inflammatory changes (acute, chronic, and recurrent inflammation), distortion, and scarring to valve dysfunction [1, 2, 5, 7].

### 4.1. Medical Treatment

Treatment of valvular manifestation of SLE depends on the type and severity of involvement. Because of the lack of large systematic studies, treatment options remain a challenge.

Important hemodynamic valvular dysfunction can be managed with conservative treatment (including ACE inhibitors, beta blockers, diuretics, immunosuppression, anticoagulation, and endocarditis prophylaxis) in some patients.

There is controversy about the place of steroids in this setting. Some investigators have suggested that the introduction of corticosteroid as the cornerstone of SLE treatment may decrease the frequency of symptoms and disease activity [4, 7, 17, 33]. Steroids do not prevent LS endocarditis, although they facilitate healing of lesions over time by decreasing the inflammation and valve damage. However, they can promote fibrosis, scarring (shortening of leaflet, chordae, and valve deformity), and thickening, resulting in additional damage and valve dysfunction [1, 4, 5, 7, 32]. There was a case report of rapid appearance of severe mitral regurgitation (by sequential echocardiography) in this SLE patient who received high dose corticosteroid therapy for acute relapse of disease [34].

### 4.2. Valve Repair

Valve surgery may be required if severe symptomatic valvular dysfunction persists [5, 7]. There is no definite consensus about surgical procedure: repair versus replacement and mechanical versus biologic valve [5, 16, 17, 32]. In current cardiac surgery, there is no doubt about the superiority of mitral valve repair versus replacement whenever possible for most cases of mitral regurgitation by different etiologies [5]. It is associated with better preservation of left ventricular function and fewer valve related complications [3].

It is suggested that mitral reconstruction might be a preferable approach as the need for anticoagulant medication is avoided. Moreover in the presence of obligatory prolonged steroid use in young patients who usually experience renal failure and undergoing hemodialysis, anticoagulation may trigger higher risk [16], especially in young females, who are likely to become pregnant in the future [5, 7].

It is believed that mitral valve repair can be justified in specific patients. If SLE status has been fairly stable with medical management and intraoperative examination shows absence of severe structural damage (with only localized and limited abnormalities), if repair seems possible and practical, then mitral valve repair is the preferred surgical option [3, 5, 16, 17]. If there is extensive fibrous network relating to papillary muscles, it may prohibit durable repair. It was reported that debridement and valve preservation would be adequate operation in the patients who had thromboembolic events from LS vegetation in the presence of normal valve structure and function [17]. In disagreement with this policy, there are case reports that show that correction of the pathologic insult for mitral regurgitation does not modify the nature of disease and valve thickening will progress; calcification and fibrosis lead to rapid recurrence of regurgitation after repair with additional risk of subsequent reoperation for replacement [4, 5, 16].

It is suggested that, in the association of SLE with hypercoagulable state like APS, repair is better option when possible [7]. APS itself increases the risk of thromboembolic complications and the need for higher intensity oral anticoagulation therapy (INR ≥ 3).

### 4.3. Valve Replacement

Valve replacement has been suggested as the choice technique because valve tissue resection en bloc will definitely prevent recurrence [35]. Mitral valve replacement with subvalvular apparatus preservation may be a good surgical alternative whenever possible [5]. But if there is fused, thick, and huge chordae and papillary muscle, complete resection is recommended as its preservation can prevent free movement of mechanical valve leaflets. Heparin should be initiated immediately after the operation to prevent thrombosis [25].

In the subject of mitral valve replacement, prosthetic valve selection is highly individualized based on age, APS association, and other conditions such as situations which need a prolonged anticoagulation use, like atrial fibrillation [3, 5, 16].

Although successful placement of biologic valve has been reported, massive thrombosis, leaflet perforation, valvulitis, and rapid mineralization were described in this situation [5, 16, 17]. In addition cases with 8 years of follow-up of biologic valve were reported; it was mentioned that in two reported long term results of biologic valve replacements were done due to pannus formation and not due to valve failure in this setting [17].

SLE associated renal failure can accelerate deterioration of the structural valve secondary to abnormal calcium and phosphate metabolism [5, 16, 18]. If renal involvement is prominent feature in SLE patient, use of mechanical valve provides better result although the risk of thromboembolic events remains. However, in patients who are at low risk with anticoagulation and high risk for biologic valve calcification, mechanical valve replacement is recommended [5].

In patients with bleeding abnormalities the superiority of biologic valves over mechanical valves is clear. If there is association of APS in SLE patient, thrombotic manifestation on the valve because of hypercoagulopathy state is prominent. It was described that thromboembolic events could occur even with a target INR of 3-4 in these patients [17]. These patients undergoing valve surgery are at higher risk of thrombotic complications and biologic valve was recommended [5, 7, 16, 17, 27].

The need for lifelong anticoagulation in mechanical valve and the risk of hemorrhagic and thromboembolic events of them despite their prolonged durability must be balanced with hypercoagulable state in APS patients and the risk of immunologic deterioration of the biologic valve (rapid calcification, valvulitis, and leaflet perforation).

### 4.4. Mitral Stenosis versus Regurgitation

It seems that SEL patients in the setting of stenosis had more adverse outcome in comparison with regurgitation. There were case reports of these patients with valve repair or replacement that had needed reoperation due to mitral restenosis [14, 15, 36]. It looks that disease progress is more prominent in stenotic valve and suggested aggressive therapy to remove vegetative tissue in the first operation as much as possible in order to prevent local extension of it that produce restenosis or malfunction of prosthetic valve. These patients may need a higher dose of steroids and immunosuppressive drugs to control the disease. Regular echocardiography and valve assessment is recommended in these patients in spite of being asymptomatic clinically. On the other hand there are reports that show mitral regurgitation symptoms and grade could be controlled frequently by medical management [16, 31, 37].

## 5. Conclusion

The experience with mitral valve surgery in SLE patients is limited. Review of the literature has shown that outcome is good in most patients who have undergone surgery but association of APS has negative impact on outcome. If there is localized abnormality in normal structure and function of mitral valve in relatively stable and controlled SLE patient, valve repair is a better choice. In patients with association of APS, due to increased risk of thrombotic events, biologic valve is advised. In active lupus nephritis and abnormal calcium metabolism, mechanical valve is a better option. In patients who are at low risk with anticoagulant, mechanical valve is suggested.

## Figures and Tables

**Table 1:** Reported cases of mitral valve repair in SLE patients.

First author	Published Year	Sex/age	Disease	Mitral pathology	Type of surgery	Outcome
Benotti [13]	1984	f/16	SLE	MR, AI	Repair of anterior mitral leaflet, AVR with biologic valve	6 y follow-up Remained significant MR

Nishimura [14]	1989	f/24	SLE	MS	MVR after 2 times OMVC	Mediastinal hemorrhage on 21st day postoperatively, died of septicemia

Pomerantzeff [15]	1999	f/48	SLE	MS	OMVC, after 6 m MVR with biologic valve	7 y follow-up

Hakim [16]	2001	f/34	SLE	MR Recurrent MR	Repair redo MVR, biologic valve	Developed CHF 1 y follow-up

Hakim [16]	2001	f/28	SLE	MR (ruptured chordae, retracted leaflet)	Repair	12 m follow-up

Gorki [17]	2008	f/27 f/53	SLE + APS SLE + APS	MS, MR MR, CAD	OMVC, Kay's annuloplasty Quadrangular resection + annuloplasty ring, CABG	18 m follow-up NR

Gorki [17]	2008	f/25	SLE + APS	MR	Repair	NR

Bouma [5]	2010	f/17	SLE	MR Developed MS after 6 months	Posterior leaflet enlargement and annuloplasty ring MVR, biologic valve	12 m follow-up

Bouma [5]	2010	f/54	SLE	MR Developed MR after 29 months	Repair MVR, mechanical valve	5 y follow-up

Bouma [5]	2010	m/23	SLE	MR	Repair	NR

Bouma [5]	2010	m/58	SLE + APS	MR	Valvuloplasty and annuloplasty	NR

Bouma [5]	2010	m/49	SLE	MR	Quadrangular resection of the posterior leaflet and annuloplasty ring	Trace MR in 11 y follow-up

Perier [18]	2011	f/NR	SLE + APS	MR	Vegetation removal, ring annuloplasty	NR

Tanawuttiwat [19]	2011	f/22	SLE	MR, AS	Vegetation removal	VF and TIA due to embolic event, 2 y follow-up

Bourré-Tessier [1]	2011	f/14	SLE	MR	Wedge resection of anterior leaflet, commisuroplasty, and posterior annuloplasty ring	10 y follow-up

Roshanali [20]	2012	f/17	SLE + RF	MS + MR	Ring annuloplasty and anterior leaflet augmentation with autologous pericardium	Need for MVR due to valve calcification in short follow-up

AI: aortic insufficiency, APS: antiphospholipid syndrome, AS: aortic stenosis, AVR: aortic valve replacement, CABG: coronary artery bypass graft, CAD: coronary artery disease, CHF: congestive heart failure, f: female, m: male, m: month(s), MR: mitral regurgitation, MS: mitral stenosis, MVR: mitral valve replacement, NR: not reported, OMVC: open mitral valve commissurotomy, RF: rheumatic fever, SLE: systemic lupus erythematous, TIA: transient ischemic attack, VF: ventricular fibrillation, and y: year.

**Table 2:** Reported cases of mitral valve replacement in SLE patients.

First author	Year	Age/sex	Disease	Mitral pathology	Type of surgery	Outcome
Reilly [21]	1988	f/NR	SLE	MS	MVR, biologic valve	24 m follow-up

Hussain [22]	1993	f/NR	SLE	MS	MVR, mechanical valve	NR

Hoffer [23] Yilmaz [24]	1999 2008	f/NR	SLE	MS restenosis	MVR, mechanical valve Redo MVR	Died in 3rd post-op dad due to cerebral hemorrhage

Hakim [16]	2001	f/29	SLE	MS (calcified, thickened leaflet)	MVR, mechanical valve	Died on second day due to renal failure

Hakim [16]	2001	m/59	SLE	MS, AI (thickened, fibrotic, and scarring)	AVR, MVR, and mechanical valve	NP

Hakim [16]	2001	f/37	SLE	MS (thickened, fibrotic) Recurrent MS (valve fixed in open position)	MVR, mechanical valve Redo MVR, mechanical valve 1 year ago	NP Died on day 4 due to valve thrombosis

Hakim [16]	2001	m/45	SLE	MS (fibrotic, calcified, and inflamed)	MVR, biologic valve	NP

Hakim [16]	2001	f/31	SLE	MS (fused chordae)	MVR, mechanical valve	NP

Hakim [16]	2001	f/16	SLE	MR (Libman-Sacks endocarditis)	MVR	NP

Hakim [16]	2001	m/30	SLE	MR, AI (ruptured chordae, muciform degeneration)	AVR/MVR, mechanical valve	NP

Hakim [16]	2001	f/40	SLE + APS	MS	MVR, biologic valve	36 m follow-up

Hakim [16]	2001	f/49	SLE + APS	Libman-Sacks endocarditis	MVR, AVR, NR	NR

Hakim [16]	2001	f/38	SLE	MR (Libman-Sacks endocarditis)	MVR, mechanical valve	NP

Hakim [16]	2001	f/66 f/40 f/25	SLE	MS (calcified, thick) MR (prolapsed leaflet) MR, AI (perforated leaflet, endocarditis)	MVR, NR MVR, NR AVR/MVR, NR	NR NR NR

Hakim [16]	2001	f/16 m/4	SLE SLE	MR (fibrotic, calcified, thick, ulcerated valve and chordate) MS (thickened, fibrotic ring and chordae)	MVR MVR	NP Died after 3 years

Hakim [16]	2001	m/44	SLE	MR (fibrotic, ruptured chordae)	MVR, NR	NR

Hakim [16]	2001	f/39	SLE + APS	MR (chordae rupture)	MVR, biologic valve	48 m follow-up

Hakim [16]	2001	f/17	SLE	MR(fused chordae, thickened valve)	MVR, mechanical valve	NR
		f/40	SLE	MR (dilated valve ring, fibrotic)	MVR, mechanical valve	Mediastinitis
		f/45		MR (fibrotic, calcified, thick, and retracted)	MVR, mechanical valve	NR

Hakim [16]	2001	f/23	SLE	MR	MVR, mechanical valve	12 m follow-up
		f/45	SLE	MR, AI (retracted, thick valve)	MVR, AVR, and mechanical valve	7 y follow-up
		f/64	SLE	MR (pink leaflet, fragile chordae)	MVR, mechanical valve	3 y follow-up

Hakim [16]	2001	f/36	SLE + APS	NR	MVR, NP	Died at 1 year, hemorrhagic CVA

Hasegawa [25]	2001	f/51	SLE + APS	MS	MVR, NR	NR

Lan [4]	2005	m/22	SLE	MR	MVR, NR	NR

Gorki [17]	2008	f/42	SLE + APS	NR	MVR, mechanical valve	NR

Gorki [17]	2008	f/29	SLE + APS	NR	MVR, mechanical valve	24 m follow-up

Gorki [17]	2008	f/41	SLE + APS	MR, MS	MVR, NR	48 m follow-up

Gorki [17]	2008	f/45	SLE + APS	NR	MVR, NR	NR

Gorki [17]	2008	f/39	SLE + APS	NR	MVR, biologic valve	108 m follow-up

Gorki [17]	2008	f/46	SLE + APS	NR	MVR, mechanical valve	NR

Gorki [17]	2008	f/31	SLE + APS	NR	MVR, mechanical valve	2 m follow-up

Gorki [17]	2008	f/48 f/43 f/38 f/51 f/54	SLE + APS SLE + APS SLE + APS SLE + APS SLE + APS	NR NR NR NR NR	MVR, mechanical valve, TV repair MVR, mechanical valve, TV repair MVR, mechanical valve MVR, mechanical valve MVR, mechanical valve	Death in 1st day Death in 22nd day 32 m follow-up 33 m follow-up 6 m follow-up

Gorki [17]	2008	f/31	SLE + APS	MR	MVR, mechanical valve	2 m follow-up

Gorki [17]	2008	f/38	SLE + APS	NR	MVR, mechanical valve	NR

Bouma [5]	2010	f/22	SLE	MR (ruptured chordae, thickened valve)	MVR, biologic valve	Alive 2 months post-op

Bouma [5]	2010	f/18	SLE	MR, MS (ruptured chordae, calcified, thickened fibrotic, scarring)	MVR, biologic valve	Alive 4 months post-op

Bouma [5]	2010	f/27	SLE	MR (ruptured chordae)	MVR, biologic valve	NP

Bouma [5]	2010	m/51	SLE	MR (ruptured chordae)	MVR, biologic valve	NP

Bouma [5]	2010	f/20	SLE	MR (fused chordae)	MVR, mechanical valve	CVA 17 months later

Bouma [5]	2010	m/43	SLE	MR (retracted, fused valve)	MVR, mechanical valve	4 y follow-up

Bouma [5]	2010	f/54	SLE	AI, MR (central perforation, vegetation)	AVR/MVR, biologic valve	26 m follow-up

Bouma [5]	2010	f/22 f/67	SLE SLE	MR (inflammatory, fibrinoid necrosis) MR (fibrotic, calcified)	MVR, biologic valve MVR, mechanical valve	NR died intraoperatively

Bouma [5]	2010	m/34	SLE	MS, AI (fibrotic, calcified, and thick)	MVR, AVR, biologic valve	30 m follow-up

Bouma [5]	2010	m/37	SLE + APS	MR, AI	MVR, AVR, and biologic valve Redo MVR after 1 y	Recurrent A-V fistula died 1 m after redoing MVR

Bouma [5]	2010	f/49	SLE + APS	NR	MVR, NR	NR

Bouma [5]	2010	f/54	SLE + APS	MR	MVR, biologic valve	Death 9 m postoperative

Bouma [5]	2010	f/44	SLE	MR	MVR, mechanical valve	6 m follow-up

Bouma [5]	2010	f/57	SLE + APS	MR	MVR, mechanical valve	MI in 2nd day, 2 m follow-up

Bouma [5]	2010	f/28	SLE + APS	MR	MVR, mechanical valve	NR

Kondo [26]	2010	f/56	SLE + secondary **amyloidosis**	MR	MVR, mechanical valve	New vegetation on replaced valve and multiorgan failure caused her death 3 m later

Bouma [5]	2010	m/56	SLE	MR	MVR, mechanical valve	18 m follow-up

Colli [27]	2010	m/37 f/32	SLE + APS SLE + APS	MR	MVR, mechanical valve MVR, biologic valve	Died on 8th day postoperative to massive ischemic stroke 5-year follow-up

Morisaki [28]	2012	f/41	SLE + APS	MS, MR	MVR	NR

Nowicka [12]	2012	f/24	SLE	MR	MVR, mechanical valve	NR

Soeiro [29]	2012	f/45	SLE + APS	MS, early structural valve deterioration, MVR (biologic valve) due to endocarditis 9 y ago	MVR, mechanical valve	NR

El-Mohamady [30]	2013	f/30	SLE + APS	MS, history of acute myocardial infarction and cerebral thromboembolic events	MVR, biologic valve	NR

AI: aortic insufficiency, APS: antiphospholipid syndrome, AS: aortic stenosis, AVR: aortic valve replacement, CABG: coronary artery bypass graft, CAD: coronary artery disease, CHF: congestive heart failure, CVA: cerebrovascular accident f: female, m: male, m: month(s), MR: mitral regurgitation, MS: mitral stenosis, MVR: mitral valve replacement, NR: not reported, OMVC: open mitral valve commissurotomy, RF: rheumatic fever, SLE: systemic lupus erythematous, TIA: transient ischemic attack, TV: tricuspid valve, VF: ventricular fibrillation, and y: year.
